# Dissecting inhibitory brain circuits with genetically-targeted technologies

**DOI:** 10.3389/fncir.2014.00124

**Published:** 2014-10-17

**Authors:** Dona K. Murphey, Alexander M. Herman, Benjamin R. Arenkiel

**Affiliations:** ^1^Department of Neurology, Baylor College of MedicineHouston, TX, USA; ^2^Program in Developmental Biology, Baylor College of MedicineHouston, TX, USA; ^3^Department of Molecular and Human Genetics, Baylor College of MedicineHouston, TX, USA; ^4^Department of Neuroscience, Baylor College of MedicineHouston, TX, USA; ^5^Jan and Dan Duncan Neurological Research Institute at Texas Children's HospitalHouston, TX, USA

**Keywords:** interneurons, optogenetics, chemical genetics, viral tracing, channelrhodopsin, interneuron diversity

## Abstract

The evolution of genetically targeted tools has begun to allow us to dissect anatomically and functionally heterogeneous interneurons, and to probe circuit function from synapses to behavior. Over the last decade, these tools have been used widely to visualize neurons in a cell type-specific manner, and engage them to activate and inactivate with exquisite precision. In this process, we have expanded our understanding of interneuron diversity, their functional connectivity, and how selective inhibitory circuits contribute to behavior. Here we discuss the relative assets of genetically encoded fluorescent proteins (FPs), viral tracing methods, optogenetics, chemical genetics, and biosensors in the study of inhibitory interneurons and their respective circuits.

## Introduction

Interneurons subserve sensory processing (Lee et al., 2012, 2013; Hamilton et al., 2013; Pfeffer et al., 2013; Fu et al., 2014), movement (Brown et al., 2014), learning (Kravitz et al., 2012), reward (Witten et al., 2010; van Zessen et al., 2012), and disease (Gradinaru et al., 2009; Kravitz et al., 2010; Krook-Magnuson et al., 2013; Peng et al., 2013; Brown et al., 2014; Cho and Sohal, 2014; Ledri et al., 2014), and genetic technologies allow for their targeted study (Taniguchi et al., 2011; Kepecs and Fishell, 2014). Various molecular, morphological, and electrophysiological properties (Ascoli et al., 2008) delimit interneuron diversity. Molecular features can be used to investigate whether cell shape or passive and active membrane properties of neurons in circuits of interest represent(s) a homogeneous class (Taniguchi et al., 2013), and morphological and electrophysiological properties of the neurons that express a characteristic marker can further elaborate within class diversity of molecularly defined populations. Additional strategies can then be used to iteratively refine molecular heterogeneity (Ma et al., 2006; Runyan et al., 2010; Chittajallu et al., 2013; Povysheva et al., 2013; Sohn et al., 2014).

Many molecular features arise from post-mitotic cell-type specification by changes in receptor expression, cell-intrinsic activity, and the activity of connected partners to modify a handful of mutually exclusive cardinal classes. Genetically targeted tools for marking and manipulating neuronal activity differentially exploit molecular expression to delimit interneuronal subtypes. In particular, the widespread application of conditionally-expressed fluorescent proteins (FPs) (Hadjantonakis et al., 2003; Livet et al., 2007), virally mediated anatomical tracers (Wickersham et al., 2007; Beier et al., 2011), optogenetic reporters (Zhang et al., 2006; Berndt et al., 2014), designer receptors exclusively activated by designer drugs (DREADD receptors) (Wess et al., 2013), and genetically-encoded subcellular biosensors (Hodgson et al., 2008; Tantama et al., 2012; Glykys et al., 2014), has allowed us dissect the contributions of specific interneurons and even their subcellular compartments to circuit function, behavior, and disease in genetically tractable mice. We discuss the benefits and challenges of these methods first with an eye to how we target the interneurons of interest for investigation.

## Strategies for genomic targeting of interneurons

Recapitulating endogenous gene expression is key to cell type-specific study (Luo et al., 2008) and can be achieved by standard, bacterial artificial chromosome (BAC) (Heintz, 2001; Ting and Feng, 2014), and site-directed transgenic methods. Standard and BAC transgenic methods both rely on random integration, which may help to study interneuron subpopulations that express the same *cis*-regulatory elements. However, site-directed integration, made possible through integrases or gene targeted knockins, more faithfully recapitulates endogenous gene expression and is therefore often preferred. Binary expression systems and/or conditional mutagenesis based on site-directed DNA recombination confer even greater experimental reliability and flexibility (Garcia-Otin and Guillou, 2006; Miyoshi et al., 2010).

It is important to note that the cell type-specificity of genetic targeting relies on accurate endogenous promoter expression. To aid in this effort there is a rapidly evolving set of comprehensive gene expression data for the mouse brain during development, and across different areas in adult brain tissue (Allen Brain Atlas, www.brain-map.org, and GENSAT, www.gensat.org). Established Cre driver lines (Kimura et al., 1996; Qiu et al., 1997; Schurmans et al., 1997; Schwaller et al., 1999; Kerr et al., 2000; Gyurko et al., 2002; Misgeld et al., 2002; Qian et al., 2002; Robledo et al., 2002; Kusakabe et al., 2006; Chattopadhyaya et al., 2007; Liodis et al., 2007; Taniguchi et al., 2011; Wang et al., 2014) (Table 1) have to date provided the greatest genetic traction on neocortical interneurons (Taniguchi, 2014) and local and long-range inhibitory neurons of the striatum (Brown et al., 2014; Nelson et al., 2014) and hippocampus (Melzer et al., 2012; Kepecs and Fishell, 2014). It is of note, however, that lineage-specific patterns of gene expression can carry into unanticipated cell types in other parts of the brain or body through early recombination events. Because only static patterns of cortical and hippocampal expression have been extensively characterized (Taniguchi et al., 2011), expression validation is critical. But, importantly, lineage effects can be co-opted by temporally restricted inducible transgenes (Rothermel et al., 2013) in order to study the developmental expression (Dymecki and Kim, 2007; Kumar et al., 2013) of cell-type-specific promoters. Complementary virus-mediated transgene expression in adult brain tissue might avoid lineage-associated issues by only targeting cell types that express a given marker at the time of viral delivery.

**Table 1:** **Useful mouse lines for commonly studied interneurons**.

**Gene name**	**Cre/Cre-ER/Tet**	**Knockout**	**Fluorescent/Functional reporter**	**References**
Agrp	Cre^Jax^, Cre-ER	Germline	N/A	Qian et al., 2002; Wang et al., 2013
Avp	Cre^Jax^	Conditional^IMSR^	GFP^MMRRC^	
Calb2	Cre^Jax^, Cre-ER^Jax^	Germline	GFP^MMRRC^	Schurmans et al., 1997
Cck	Cre^Jax^, Cre-ER^Jax^	Germline^Jax^	GFP^MMRRC^	
Chat	Cre^Jax^, Cre-ER^Jax^	Germline, Conditional^Jax^	GFP^Jax^, Chat-Chr2::eYFP^Jax^	Misgeld et al., 2002
Crh	Cre^Jax^	Germline^Jax^	GFP^MMRRC^	
Dlx1	Cre^MMRRC^, Cre-ER^Jax^	Germline	tdTomato^MMRRC^	Qiu et al., 1997
Dlx5/6	Cre^Jax^, Cre-ER^Jax^	Germline	GFP^Jax^	Robledo et al., 2002
Gad1 (Gad67)	Cre	Germline^Jax^, Conditional	GFP^Jax^	Chattopadhyaya et al., 2007; Taniguchi et al., 2011
Gad2 (Gad65)	Cre^Jax^, Cre-ER^Jax^	Germline^Jax^	GFP^MMRRC^, mCherry^Jax^	
Gal (Galanin)	Cre^MMRRC^	Germline	GFP^MMRRC^	Kerr et al., 2000
Lhx6	Cre-ER^Jax^	Germline	GFP^MMRRC^	Liodis et al., 2007
Nos1	Cre^Jax^, Cre-ER^Jax^	Germline^Jax^, Conditional	GFP^MMRRC^	Gyurko et al., 2002
Nkx2.1	Cre^Jax^, Cre-ER^Jax^	Germline, Conditional	GFP^MMRRC^	Kimura et al., 1996; Kusakabe et al., 2006
Pvalb	Cre^Jax^, Cre-ER^Jax^, Tet-Off^Jax^	Germline	GFP^MMRRC^, Pvalb-Chr2::eYFP^Jax^	Schwaller et al., 1999
Slc32a1 (Vgat)	Cre^Jax^, Cre-ER^Jax^	Germline^Jax^, Conditional^Jax^	GFP^MMRRC^, Vgat-Chr2::eYFP^Jax^	
Sst	Cre^Jax^, Cre-ER^Jax^, Tet-Off^Jax^	Germline^Jax^	N/A	
Npy	Cre^MMRRC^, Tet-Off^Jax^	Germline^Jax^	GFP^Jax^	
Vip	Cre^Jax^	Germline^Jax^	GFP^MMRRC^	

*Availability index: Jax, Jackson Laboratory; MMRRC, Mutant Mouse Regional Resource Center; IMSR, International Mouse Strain Resource*.

## Viral delivery of genetically encoded tools in interneurons

Restriction of a reporter to a subset of neurons in a given brain area or developmental time point can be achieved by viral delivery of genetic constructs driven by cell type-specific promoters. Viral delivery of transgenes driven by short promoters (Nathanson et al., 2009) has not effectively captured a genetically homogeneous subpopulation of neurons, but conditional Cre-dependent [e.g., Flip Excision (Flex) (Schnutgen et al., 2003) or Lox-Stop-Lox] transgenes driven by strong ubiquitous promoters segregate neurons spatially and temporally in a way not possible through genomic strategies. Viral injections are constrained by their physical properties, however. Viral genome size limits the size of the genetic constructs viruses can carry, and efficiency of uptake and direction of infectivity (Rothermel et al., 2013) depend on viral capsid serotype, genetic construct (Betley and Sternson, 2011), and the targeted brain region (Taymans et al., 2007). Controls should ensure that successful viral infection does not alter cell function. Acknowledging these caveats, gene targeting through both conventional strategies, and virus-mediated transgene delivery, offer an increasingly powerful reserve of anatomical and functional tools we highlight below.

## Genetically encoded anatomical dissection of interneurons

Interneurons assume diverse somatic shapes and patterns of dendritic/axonal arborization in cortical (Markram et al., 2004; Ascoli et al., 2008) and subcortical structures (Petryszyn et al., 2014). Certain morphologies tend to elaborate certain electrophysiological properties, but there is both variability and overlap of form and function. While some fundamental features are conserved within classes (Rudy et al., 2011; Pfeffer et al., 2013), we still find notable heterogeneity among even well-described groups (Ma et al., 2006; Runyan et al., 2010; Chittajallu et al., 2013; Povysheva et al., 2013; Sohn et al., 2014). Dissecting this heterogeneity has become tractable with genetically-encoded FPs (Hadjantonakis et al., 2003; Livet et al., 2007; Kremers et al., 2011) whose cell-fill and membrane-directed expression has allowed for real-time visualization of interneurons in intact functional preparations such as juxtacellular recording or calcium imaging (Tukker et al., 2007; Kato et al., 2013; Chiovini et al., 2014; Lee et al., 2014) as well as more precise surveys of circuit connectivity. FPs have also been useful for certain neuropeptidergic subclasses of interneurons such as SST and VIP, in which the extensive processing of propeptides and the extra-somatic localization of their products have made these cell types difficult to visualize using conventional immunohistochemistry (Nassel, 1993). It is important to recognize that FPs exhibit variable stability (turnover, photo/pH/temperature stability) subject to differential regulation in each cell type. To better characterize cell shape and connectivity, however, genetically targeted FP expression offers a good promontory. The use of neurotropic viruses further reveals circuit connectivity of specific cell types.

Neurotropic viruses selectively infect neurons. Polysynaptic, monosynaptic, anterograde, and retrograde transport of viral vectors for cell labeling are now possible (Kuypers and Ugolini, 1990; Zemanick et al., 1991; Enquist and Card, 2003). One of the first viral tracing vectors to be implemented was modified herpes simplex virus (HSV), which shows both anterograde and retrograde transport and has recently been engineered to be cre-dependent for greater cell type-specificity (Lo and Anderson, 2011). HSV can be used to establish the polysynaptic connectivity of a circuit, but this viral tracing approach has been limited by cytotoxicity. Emergent methods for genetically-encoded monosynaptic viral tracing (Wickersham et al., 2007; Beier et al., 2011) now offer more sophisticated alternatives toward dissecting neuronal circuits. Using genetically engineered rabies virus (RV), alongside pseudotyping and cell type specific targeting approaches (Wall et al., 2010; Weible et al., 2010), we can now safely query monosynaptic inputs onto cells. Moreover, RV can encode dual fluorescent and functional reporters, such that monosynaptic pairs can be visualized and manipulated dynamically (Osakada et al., 2011).

As powerful as the new viral vectors are toward revealing brain connectivity, interpreting genetically targeted monosynaptic tracing studies in interneuronal circuits can be vexing. Interneurons, more so than principal excitatory neurons, promiscuously synapse onto other inhibitory cells in order to exert disinhibitory control in development (Kuhlman et al., 2013) and in the adult brain (Lee et al., 2013; Pfeffer et al., 2013; Pi et al., 2013; Xu et al., 2013). Reciprocal intraclass chemical synapses and/or electrical gap junctions coordinate activity across large populations of neurons (Tamas et al., 1998, 2000; Chiu et al., 2013; Hioki et al., 2013) and over considerable distances (Buzsaki et al., 2004; Caputi et al., 2013), confounding the identification of presynaptic-postsynaptic partners. Polysynaptic tracers and functional tools can be further implemented to clarify connectivity.

## Genetically encoded functional dissection of inhibitory circuits

Recently, the light-activated non-specific cation channel channelrhodopsin-2 (ChR2) and its variants (Mattis et al., 2012) have been extremely valuable toward unraveling fundamentals of functional connectivity both *ex vivo* and *in vivo* (Huang et al., 2013; Jennings et al., 2013; Stamatakis et al., 2013; Halassa et al., 2014; Roux et al., 2014; Siegle and Wilson, 2014; Sparta et al., 2014). However, the interpretation of optogenetic manipulations in inhibitory neurons must be approached with some caution given the diversity of cell type-specific responses to photic stimulation. For example, using varying light stimulation parameters to activate different ChR2-expressing cell types, we learned that cortical somatostatin-positive interneurons exhibit heterogeneous firing properties, and that all regular-spiking interneuron subtypes evaluated including somatostatin, corticotropin-releasing hormone (CRH), and cholinergic interneurons could be functionally silenced, rather than activated, when photically stimulated with prolonged light pulses (Herman et al., 2014). An appropriate strategy would be to first identify the effect of stimulation on the cell type of interest using intracellular recordings or imaging techniques in order to avoid confounding interpretations at the level of post-synaptic electrophysiological probes and/or behavioral readouts. Any differential responses to optogenetic activation may then help to further subclassify interneurons.

As an alternative, or in parallel with optogenetic activation by ChR2, inhibition through light-activated chloride pumps (halorhodopsins) (Gradinaru et al., 2008; Tye et al., 2011) or proton pumps (archaerhodopsins) (Madisen et al., 2012; Beppu et al., 2014) allows us to query the direct circuit effect of temporally precise neuronal silencing. Whereas the light-gated activators have been relatively robust to engineering, the inhibitors have required ongoing reengineering to address issues such as intracellular accumulation/aggregation (halorhodopsins) and limited hyperpolarization due to proton pump kinetics (archaerhodopsins). Recently, two different groups developed an appealing alternative to inhibitory pumps by site-directed mutagenesis of channelrhodopsin, transforming it into a chloride-conducting channel. Notably, inhibitory channels have proven to be more efficient than ion pumps due to independence from photon-gated movement of individual ions, and preservation of normal electrochemical gradients (Berndt et al., 2014; Wietek et al., 2014). Reversibly silencing inhibitory interneurons can be quite useful with our growing knowledge of the behavioral contingencies that determine interclass activity differences (Letzkus et al., 2011; Lapray et al., 2012; Pi et al., 2013), as well as their differential role in network oscillations (Roux et al., 2014). This genetically targeted manipulation can also be used in the study of diseases with an evolving dysfunction of specific interneuronal cell types (Gernert et al., 2000, 2002; Kalanithi et al., 2005; Kataoka et al., 2010; Gittis et al., 2011; Kim et al., 2014), particularly to examine trial-by-trial, or time-locked variability in electrophysiology and behavior in the disease state.

Given the timescale at which optogenetic reporters function, they are appropriate for manipulating rapid, time-sensitive circuit properties that influence behaviors. However, for behaviors or disease states that require changes in activity to persist over longer intervals, chronic photic activation or inhibition of neurons can be technically cumbersome and even deleterious to the cells under investigation. If the scientific question requires persistent activity manipulation in a population of neurons to influence behaviors occurring over larger timescales, pharmacologically-activated designer receptors offer an alternative approach (Wulff and Arenkiel, 2012). Transgenic overexpression of an endogenous ionic receptor can be modulated by application of its ligand to produce membrane depolarization (Drenan et al., 2008; Kim et al., 2012), but interactions with the native ligand-receptor pair might unpredictably influence experimental outcomes. Chemically and genetically engineered ligand gated ion channels (Wulff et al., 2007) insensitive to endogenous ligands (Wulff et al., 2007; Magnus et al., 2011; Sternson and Roth, 2014), or mammalian expression of channel proteins or excitatory or inhibitory G protein-coupled receptors (GPCRs) from invertebrates (Lechner et al., 2002; Slimko et al., 2002) strategically attempt to avoid this confound, but engineered GPCRs offer perhaps the most elegant alternative (Armbruster et al., 2007). A recent incarnation of these is the DREADDs (designer receptors exclusively activated by designer drugs) (Armbruster et al., 2007), which employ an engineered receptor-synthetic ligand pair that is completely orthogonal to its endogenous equivalent, exhibits little or no baseline activity, and allows for genetically targeted activation or inhibition (Ferguson et al., 2011; Krashes et al., 2011; Ray et al., 2011). GPCRs mediate intracellular signaling cascades activated by various monoaminergic neurotransmitters and neuropeptides, more faithfully recapitulating the postsynaptic changes that may ensue with activation or inhibition of interneuronal cell types. As many neuropsychiatric disorders are the result of dysfunction or loss of these interneurons, designer GPCRs may also generate conditions that most resemble disease states. Furthermore, they offer the unique advantage of functionally dissecting intact deep subcortical circuits in a way that is more difficult or not possible with optogenetic and imaging methods used readily at the cortical surface. In order to study the complex compensatory changes that may occur with chronic, irreversible cell type-specific loss, we can employ genetically targeted lesions (Buch et al., 2005). Both reversible ligand-mediated methods and genetic lesions offer the advantage of being minimally invasive, and require only viral delivery of a receptor and peripheral application of its cognate ligand. These techniques can also be used concurrently with electrophysiological recordings without introducing overt artifacts, though a remarkable new alternative enables cross-talk free all-optogenetic actuation and indication using genetically modified channelrhodopsins and archaerhodopsins (Hochbaum et al., 2014) as well.

Genetically encoded molecular indicators augment the information that is available through traditional electrophysiological and behavioral assays of circuit function. These biosensors come in many varieties; briefly discussed here are fluorescent indicators for calcium, chloride, and neurotransmitter vesicular release. Calcium biosensors come in two forms—single wavelength sensors and ratiometric sensors that rely on fluorescence energy resonance transfer (FRET). GCaMP, an example of the former, is a fusion of GFP with calmodulin, an intracellular protein that binds calcium. In the presence of calcium, calmodulin undergoes a conformational change, which renders the GFP brighter than at baseline (Tian et al., 2009), thus allowing the detection of increased neuronal activity that occurs with calcium entry. This tool fundamentally relies on measurements of relative fluorescence. FRET allows for more stable and high resolution long term imaging, which can be used to study the neuromodulatory role of interneurons in fluctuating brain states as well as subcellular localization of GPCR signaling (protein kinase A) by neuromodulators (Chen et al., 2014). Interneurons tend to have characteristic projections onto the soma, dendrites, and axons of postsynaptic target cells, subcellular compartments that can be resolved by this technique. Ratiometric measurements can also differentiate resting state Ca^2+^ from Ca^2+^ influx of tonically firing neurons, a property that also segregates differentially between and within interneuronal classes. While calcium indicators have been especially useful to study neuronal activity (Thestrup et al., 2014), this reflects only the spiking behavior of the interneurons rather than their network level inhibitory outputs. A complementary approach to study inhibition takes advantage of the genetically encoded fluorescent chloride indicator, clomeleon (Kuner and Augustine, 2000). Clomeleon can be used to study network effects of GABA activity, and uniquely, developmental neuronal changes in Cl^−^ to report neuronal inhibition. Finally, synaptopHluorin is a genetically encoded neurotransmitter indicator engineered as a fusion between synapsin, a presynaptic vesicular fusion protein, and a pH sensitive GFP. In an acidic environment of synaptic vesicles prior to release, the GFP does not fluoresce. Alkalinization of the vesicles upon release into the extracellular space with neuronal activity results in bright fluorescence. Together, each of these neuroimaging tools allows for insight into the circuit connectivity of interneurons.

## Conclusions and directions for future research

Interneurons exhibit heterogeneous origins, morphologies, and functions that render their detailed characterization challenging. We advocate for an intersectional dissection of this heterogeneity. Starting with genetic and molecular tools (Table 2), we can characterize cardinal groups of interneurons that we further parse with powerful new techniques such as single cell gene expression analyses (Kamme et al., 2003) to elaborate iteratively what molecules better define classes. Though we did not expand on functionally characterizing interneurons with an eye to their role in specific behaviors (Kvitsiani et al., 2013; Courtin et al., 2014) and their dysfunction/attrition in disease (Verret et al., 2012; Benarroch, 2013; Del Pino et al., 2013), this may also help to constrain their undeniably vast genetic and molecular diversity. As interneuron classes are delimited operationally, and dynamically refined using genetically targeted methods, answers emerge about the shared molecular features of specific neuronal subsets that can then be exploited to generate targeted genetic approaches to their further study.

**Table 2:** **Common genetically-targeted technologies in neuroscience research**.

			**Strengths**	**Challenges**	**References**
Anatomical	Genetically-encoded fluorescent proteins	GFP, RFP, BFP, etc.	Can visualize soma or projections, can be tagged with functional reporters or overexpression constructs, real time visualization, many variants	Stability (turnover, photo/pH/temperature stability), fluorescent tags may affect tagged protein's function	Hadjantonakis et al., 2003; Livet et al., 2007; Kremers et al., 2011
	Genetically-encoded viral tracing	Rabies	Neurotropic, retrograde propagation, may be pseudotyped for infection selectivity, can reveal polysynaptic or monosynaptic connectivity, can be combined with functional reporters, high expression	Cytotoxicity (limits experimental time frame), nascent anterograde tracing strategies, can be used in concert with functional reporters, polysynaptic tracing cannot distinguish first-order connectivity	Wickersham et al., 2007; Wall et al., 2010; Weible et al., 2010; Beier et al., 2011; Osakada et al., 2011
		HSV	Cre-dependent variants available for specificity, polysynatpic tracing, retrograde and anterograde varieties available	Very high cytotoxicity (limits experimental time frame) limiting its use with functional reporters, polysynaptic tracing cannot distinguish first-order connectivity	Kuypers and Ugolini, 1990; Zemanick et al., 1991; Enquist and Card, 2003; Lo and Anderson, 2011
Functional	Genetically-encoded optogenetics	Excitatory Channelrhodopsin	Precise temporal control, can be used to directly assess functional connectivity, can be used *in vitro*/*ex vivo*/*in vivo*/awake, many variants with differential photo-kinetics	Potential for channel desensitization or depolarization block, not always sufficient for *in vivo* chronic activation experiments, physiologically relevant for excitable cell types more than for non-excitable cell types	Zhang et al., 2006; Mattis et al., 2012; Hochbaum et al., 2014
		Inhibitory Channelrhodopsin	Precise temporal inhibition, independent of photon-gated ion movement, more physiologic	Dependence on external pH	Berndt et al., 2014; Wietek et al., 2014
		Halorhodopsin	Precise temporal inhibition	Subcellular trafficking issues in older variants	Gradinaru et al., 2010; Tye et al., 2011
		Archaerhodopsin	Same as for halorhodopsins but new variants hyperpolarize more, can be used to manipulate pH, can be used as an actuator as well as an indicator	Limited by proton pump kinetics often requiring continuous photostimulation, older variants dim with long time constants and photocurrents	Madisen et al., 2012; Beppu et al., 2014; Hochbaum et al., 2014
	Genetically-encoded chemical genetics	nAchR, TRPV1	Endogenous receptor expression and ligand application to most faithfully recapitulate neuronal activation, timescale of activation within seconds	Unpredictable interactions with the native ligand-receptor pair, baseline depolarization in the absence of ligand	Drenan et al., 2008; Kim et al., 2012
		Interspecies channel proteins or GPCRs	Ligand-receptor selectivity	G-protein-coupled receptor off-target effects, ligand usually does not cross blood-brain barrier and thus must be applied locally using invasive procedures	Lechner et al., 2002; Slimko et al., 2002
		GABAA	Single modified endogenous GABAA receptor can be agonized and antagonized by different ligands (zolpidem, DMCM, respectively)	Requires a genetically-engineered zolpidem-insensitive background	Wulff et al., 2007
		PSEM	Ligand-receptor selectivity, are not GPCR-based and thus minimize G-protein-coupled off-target effects, non-invasive ligand administration	May not be adequate for sustained activation/inhibition over prolonged periods compared to other methods (e.g., DREADDs).	Magnus et al., 2011; Sternson and Roth, 2014
		DREADD	Minute-hour activation/inhibition, manipulates excitable and non-excitable cells, recapitulates dysfunction in disease, non-invasive ligand administration	G-protein-coupled receptor off-target effects, requires different receptors for activation versus inhibition	Ferguson et al., 2011; Krashes et al., 2011; Ray et al., 2011; Wulff and Arenkiel, 2012; Wess et al., 2013
	Genetically-encoded molecular imaging	GCAMP	*In vivo* imaging of neuronal activity, continually re-engineered for improved signal to noise	Limited dynamic range, indirect measure of action potentials, cannot parse resting from tonic activity, trade-off between Ca^2+^-binding affinity and response	Tian et al., 2009
		Twitch (FRET-based)	More stable long-term *in vivo* imaging, brighter, subcellular visualization, differentiates resting state Ca^2+^ from tonic firing, large dynamic range, linear responses	Indirect measure of action potentials, trade-off between high calcium binding affinity and response kinetics.	Thestrup et al., 2014
		Clomeleon (FRET-based)	Can be used to study developmental neuronal changes in Cl^−^ as well as network effects of GABA activity	Lower affinity (~ 30 mM) compared to the intracellular Cl^−^ concentration (~10 mM); pH sensitive, photobleach at different rates, interfering with FRET signal	Kuner and Augustine, 2000; Glykys et al., 2014
		SynaptopHluorin	Measures the release of neurotransmitters	Diffusional loss of the reporter	Granseth et al., 2006

### Conflict of interest statement

The authors declare that the research was conducted in the absence of any commercial or financial relationships that could be construed as a potential conflict of interest.
